# Various Recipes

**Published:** 1856-04

**Authors:** 


### VARIOUS RECIPES.

Cleaning Windows.—The neatest thing for cleaning windows or glass ware is a piece of deer skin or leather that is soft and somewhat fuzzy. Leather is better than cloth because no particles of lint or dirt will come off to adhere to the glass. A hand-basin is plenty large enough for washing windows. The great splashing some folks make in doing the work is indeed useless—it is more than useless, positively injurious. For when the waler runs in copious floods over the windows, it affects lhe putty with which the glass is set, and loosens it; besides it has a tendency to stain it, or at least to leave it dingy and unclean.

Two pieces of wash leather and a basin of suds is all that is necessary. Use the one to wash the glass with in the suds, and the other dry to wipe it with. The dry one should not be applied till the glass becomes nearly or quite dry, when a good rubbing will clean it effectually.

---

Pickles.—Pick over the cucumbers, and reject all that are broken or bruised, for they will injure the rest. Make a strong brine, and cover them with it three or four days, turning them up from the bottom every morning. Then scald vinegar enough to cover them, with whole pepper, cinnamon and mustard seed, a tablespoonful of each to one and a half gallons of vinegar. Take the cucumbers from the brine, drain them or dry them on a cloth, pack them in the jar that is to be used, and pour the vinegar boiling hot over them. Let them remain two days; pour off the vinegar, scald it again, adding a piece of alum the size of a hickory nut, to make them crisp. The best cider vinegar should be used. After two days break one open, and, if not greened through, scald them again. If well covered, they will keep for years, and grow better.

---

Glass Stoppers.—When the glass will not come out, pass a strip of woolen cloth around it, and then "see-saw" backwards and forwards, so that the friction may heat the neck of the bottle. This will cause it to expand, become larger than the stopple, and the latter will drop out or may easily be withdrawn. A tight screw may be easily loosened from a metal socket, by heating the latter by means of a cloth wet with boiling water, or in any other way—or the simple principle of expansion by heat.

---

Bread.—One of the most important household rules is, not to eat new bread, for it is expensive and unwholesome, and does not afford near so much nourishment as bread two or three days old.

